# Test–retest reliability of the Test of Gross Motor Development-3 instrument for children with Down syndrome

**DOI:** 10.4102/ajod.v15i0.1925

**Published:** 2026-04-22

**Authors:** Seyide Abiodun-Salawu, Pieter H. Boer

**Affiliations:** 1 Department of Physical Activity, Sport and Recreation, Faculty of Health Sciences, North-West University, Potchefstroom, South Africa; 2 Department of Human Movement Science, Faculty of Education, Cape Peninsula University of Technology, Wellington, South Africa

**Keywords:** ball skills, locomotor skills, motor performance, minimal detectable change at the 95% (MDC_95_), reliability

## Abstract

**Background:**

The Test of Gross Motor Development-3 (TGMD-3 2019) evaluates fundamental gross motor skills across two domains: locomotor and ball skills.

**Objectives:**

The purpose of this study was to determine the test–retest reliability of the TGMD-3 in children with Down syndrome.

**Method:**

Twenty-four children with Down syndrome, aged 9–15 years, from five special needs schools in the North West province of South Africa participated in this study.

**Results:**

Excellent and good intraclass correlation coefficient values were reported for locomotor (0.91), ball (0.84) and overall gross motor performance (0.91). Standard error of measurement, minimal detectable change at the 95% and Bland–Altman plots showed acceptable precision levels, low variability, a small discrepancy between scores for the two assessments and no systematic bias in the analyses. The test and retest values for locomotor (*p* = 0.587), ball (*p* = 0.403) and overall gross motor scores (*p* = 0.321) were not significant.

**Conclusion:**

The TGMD-3 instrument showed good to excellent test–retest reliability for assessing gross motor skills in children with Down syndrome.

**Contribution:**

This study provides an initial reliability analysis of the TGMD-3 instrument amongst selected children with Down syndrome in five districts of the North West province of South Africa.

## Introduction

Conducting a comprehensive evaluation of children’s motor abilities is crucial for understanding their developmental paths and delivering focussed assistance when required (Gu et al. 2021; Jones et al. 2020). The assessment of motor abilities of children can aid in the early detection of developmental delays, both in the intellectual and psychological domains (Faruk et al. 2020; Scheuer, Herrmann & Bund 2019). This makes the motor skill assessment an important constituent of programmes in various fields and professions, such as special needs education, occupational and physical therapy, paediatrics, neurology and rehabilitation medicine (Faruk et al. 2020). Children with well-developed coordination participate more effectively in sports and physical education sessions. However, this is not the case for children with intellectual disabilities who exhibit poorer gross motor performance than typically developing children (Özkan & Kale 2023). Furthermore, studies report that children with Down syndrome not only exhibited weaker performance compared to that of typically developing children, but also those with borderline intellectual functioning on locomotor and ball control tasks, as assessed with the Test of Gross Motor Development (TGMD)-2 (Alesi et al. 2018).

Unfortunately, not much is known regarding the cognitive developmental trajectories of children and adolescents with Down syndrome, where norm-referenced standards according to developmental age are scarce (Patterson, Rapsey & Glue 2013). What is known is that the gross motor performance of children with Down syndrome shows delayed motor and mental development, which could be associated with structural impairments in the neurological system, slower and lagged brain development and an impaired somatosensory system (Horvat, Croce & Fallaize 2016; Malak et al. 2015; Patterson et al. 2013; Volman, Visser & Lensvelt-Mulders 2007). Consequently, individuals with Down syndrome demonstrate motor learning difficulties and require consistent practise and repetition to attain proficiency in gross motor skills (Kakejani et al. 2025; Valentini & Rudisill 2004).

Volman et al. (2007) reported that the functional limitations studied in children with Down syndrome can be improved, since the impairment in motor capacity has a greater association with functional ability than mental aptitude. Whilst this impaired gross motor proficiency may be attributed to the many health-related conditions that these individuals are born with (Capone et al. 2018; Foley & Killeen 2019), the weaker gross motor performance could also be related to this population’s sedentary lifestyle (Diaz 2020), high prevalence of obesity (Bertapelli et al. 2016) and the very low physical activity levels (Fox et al. 2019). Conditions such as intellectual disability and autism spectrum disorder are neurodevelopmental disabilities (Downs et al. 2020); furthermore, intellectual disability is characterised by impairments in intellectual and adaptive functioning (Pratt & Greydanus 2007; World Health Organization 2019). For these reasons, the low muscle tone and muscle strength (Carmeli et al. 2002; Mendonca, Pereira & Fernhall 2010) reported in most individuals with Down syndrome do not benefit their neurologically coordinated movements as evidenced, for example, by their impaired balance (Villamonte et al. 2010). However, recent studies report that the gross motor skills and various physical and functional components of individuals with Down syndrome can be improved with structured exercise training (Staples, Pitchford & Ulrich 2020).

The TGMD-3 instrument is widely used to assess the gross motor performance of children (Ulrich 2000) and consists of 13 gross motor skills frequently taught in Physical Education classes (Webster & Ulrich 2017). The skills are grouped into two subtests, each of which assesses a different aspect of gross motor development (locomotion and object control). The psychometric properties of the TGMD instrument have not only been tested in typically developing children (Webster & Ulrich 2017, [TGMD-3]), but also in children with disabilities such as autism spectrum disorder (Allen et al. 2017, [TGMD-3]), developmental coordination disorder (Roczniak et al. 2025, [TGMD-3]), intellectual disability (Capio, Eguia & Simons 2016, [TGMD-2]; Simons & Eyitayo 2016, [TGMD-3]; Simons et al. 2008, [TGMD-2]), Down syndrome (Capio et al. 2018, [TGMD-2]; Tun et al. 2023, [TGMD-2]) and visual impairment (Houwen et al. 2010, [TGMD-2]). Health professionals, physical therapists, physical educators and researchers require field-based assessments of gross motor competence that are valid, reliable and feasible in order to provide them with clinically relevant information pertinent to decision-making purposes. There are also other well-known, standardised and available instruments that assess motor skills, such as the Bruininks-Oseretsky test of motor proficiency-2 (BOT-2) (Bruininks & Bruininks 2005) and the movement assessment battery for children-2 (MABC-2) (Henderson, Sugden & Barnett 2007). However, the domain of measurement is different, focussing on fine manual control, body coordination, strength and agility (BOT-2), manual dexterity, ball skills, aiming and catching and static and dynamic balance (MABC-2) (Baharudin, Harun & Kadar 2020). Thus, the main focus of these instruments is not overall gross motor skills, although specific gross motor tests are included. Furthermore, the BOT-2 assessment requires 45 min – 60 min of testing per person and a significant amount of space, five practise and 10 testing trails, and that specific equipment from the manufacturer be used (Baharudin et al. 2020; Piek, Hands & Licari 2012; Spironello et al. 2010), additionally, the MABC-2 does not cover the full range of motor ability testing.

Reliability, as an important psychometric property of a standardised instrument, is a pre-requisite for the educational, clinical and research application of any given measure even more so for field-based measures such as the TGMD-2 and 3 (Rey et al. 2020). Reliability is the measure of the consistency and stability of an instrument when subjected to repeated applications. In order to reliably measure consistency and change in motor skill development over time in assessment, a reliable instrument is needed for the specific population being tested. Without a reliable instrument, variations in testing after an exercise intervention may stem from equipment or procedural inconsistencies rather than genuine advancements in motor ability (Simons et al. 2008). In addition, reliability is essential for ensuring fair and accurate evaluations, most importantly for children with disabilities such as autism spectrum disorder, intellectual disability and Down syndrome. A reliable and consistent assessment instrument ensures the accurate measurement of motor abilities that are devoid of inconsistencies or technical errors (Brian et al. 2018). In addition, a reliable instrument also ensures the safety and effectiveness of physical activity assessments (Simons et al. 2008).

The reliability of the TGMD-3 has been discussed extensively by various researchers (Maeng, Webster & Ulrich 2016; Pitchford & Webster 2020; Ulrich 2019; Webster & Ulrich 2017). The recent systematic study of Rey et al. (2020) further explored the reliability of the instrument in various child and adolescent populations using the second and third editions of TGMD (Rey et al. 2020). Another recent systematic review conducted specifically for children with intellectual disability and autism spectrum disorder also reported on the various reliability and validity coefficients of a range of gross motor performance instruments, including the TGMD-2 and 3 and Bruininks-Oseretsky Test of Motor Proficiency (Downs et al. 2020).

Although the test–retest reliability of the TGMD-2 instrument has been performed on children with intellectual disability (Simons et al. 2008) and Down syndrome (Tun et al. 2023), it remains to be determined for the TGMD-3, as the study by Allen et al. (2017) was conducted on children with autism spectrum disorder. Even though the study by Tun et al. (2023) was conducted on children with Down syndrome, the researchers did not provide evidence of measurement error, minimal detectable change or systematic bias. Furthermore, the study reported significant improvements in gross motor performance from test to retest. Due to the diminished and often delayed motor and mental development of children with Down syndrome, the test–retest reliability of the revised TGMD-3 instrument should be assessed for reliability coefficients, measurement error and bias indices. Hence, the purpose of this investigation was to determine the test–retest reliability of the newest edition of the TGMD-3 in measuring motor proficiency amongst children with Down syndrome.

## Research methods and design

### Participants

Twenty-four children with Down syndrome, 13 boys and 11 girls, participated in this study. Participants were identified from five special needs schools in the North West province of South Africa and were between 9 years and 15 years of age. A summary of their demographic characteristics is provided in Table 1. A purposive sampling approach was applied, wherein all children with Down syndrome in the five special needs schools were afforded the opportunity to participate in the study. Participation in the study was entirely voluntary.

**TABLE 1:** Demographic characteristics of study participants.

Characteristics	Frequency	%
**Age (years)**		
9–11	14	58
12–15	10	42
**Gender**		
Boys	13	54
Girls	11	46
**Race**		
African people	20	84
Mixed-race people	2	8
White people	2	8
**Total**	**24**	**100**

### Procedures

Before the study commenced, all completed consent and assent forms were collected from the participants and their parents and/or guardians through the administrators in the special needs schools. The researcher completed the first section of the TGMD-3 examiner record form, which contains the participants’ identifying information (name, school and age). Pseudonyms were used for the participants on the recording form, which ensures anonymity. All data were gathered in the respective school halls, which ensured a safe and non-slippery surface and an environment free from noise and disruptions.

Participants were tested on two occasions at a 1-week interval (Webster & Ulrich 2017). One week after the first test, the participants were tested again at the same time of day, using the same procedure and order of testing as during the initial assessment. Ulrich (2019) and Webster and Ulrich (2017) also used a 1-week testing interval when assessing test–retest reliability.

All testing procedures were applied as outlined in the testing manual of the TGMD-3 instrument (Ulrich 2000). Two participants were examined at a time. The locomotor subtests were assessed first, followed by the ball skills subtest. The 13 tests included a short run, gallop, hop, skip, horizontal jump, slide, two-handed strike of a stationary ball, forehand strike of a self-bounced ball, one-hand stationary dribble, two-handed catch, kick of a stationary ball, overhead throw and underhand throw. The researcher assigned one if the skill was performed correctly and zero if it did not meet the criterion. Testing lasted approximately 30 min per pair of participants. An example of the 13 tests and the equipment that was required is shown in Online Appendix 1.

The researcher, who is an adapted physical activity specialist and a trained administrator, performed a physical demonstration and provided a verbal explanation of each test before the participants were allowed a practice trial prior to the assessment (as outlined in the TGMD-3 manual) (Ulrich 2019).

### Measuring instrument

The reliability and validity of the TGMD-3 instrument have been determined and confirmed in 807 typically developing children (Webster & Ulrich 2017). The researchers reported excellent test–retest reliability coefficients for the locomotor (0.97), ball (0.95) and total gross motor scores (0.97). The TGMD-3 instrument consists of six locomotor skills (run, gallop, hop, skip, horizontal jump and slide) and seven ball skills (two-hand strike of a stationary ball, one-hand forehand strike of a self-bounce ball, one-hand stationary dribble, two-hand catch, kick of a stationary ball, overhand throw and underhand roll), as reported in Ulrich (2019).

Although the TGMD-3 instrument was designed for children of up to 10 years of age, its use for older children is also reported in other studies of intellectually disabled children and adolescents (Magistro et al. 2018; Zhang, Wang & Wu 2024). Furthermore, studies conducting reliability analysis for children with developmental coordination disorder (Roczniak et al. 2025), intellectual disability (Capio et al. 2016) and Down syndrome (Capio et al. 2018) also tested children up to 14 years of age. The suitability of TGMD-3 for the evaluation of children with developmental disorder that are above 10 years old can be ascribed to their developmental delays of 3–5 years, compared to typically developing children (Capio et al. 2016; Simons et al. 2008).

**FIGURE 1:**
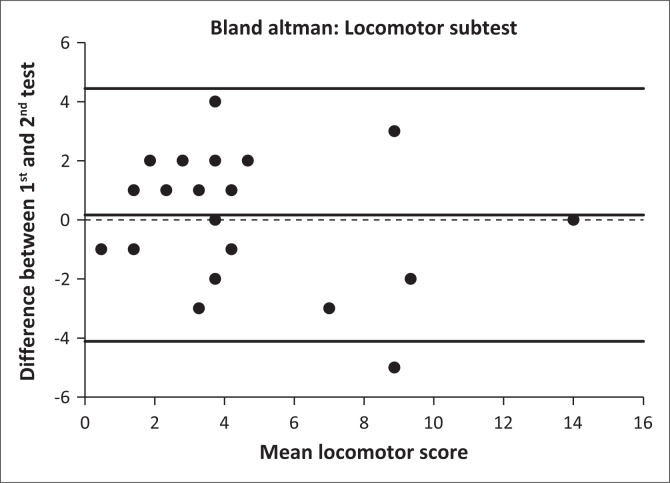
Bland–Altman plot for the locomotor subtest.

The skills were evaluated using certain performance criteria for each one of the 13 tasks. The minimum performance criteria for some of the tests is three and the maximum is five. For example, in the two-handed catch ball skill, the performance criteria included: (1) the child stretches hands out in front of the body with elbows bent, (2) the child stretches hands for the ball, and (3) the child catches the ball with hands only. Each of the 13 gross motor skills is assessed on two trials (excluding familiarisation practice trials). For each trial, a performance score of either zero or one is awarded: zero for incorrect skill demonstration and one for correct skill demonstration. The scores from both trials were added to calculate the total score for that specific item. The addition of all locomotor and ball skill scores provided the total raw score for each subtest, locomotor and ball skills, respectively. The total raw scores for locomotor ability range from 0 to 46, whereas the total raw scores for ball ability range from 0 to 54. Consequently, total gross motor scores fall within a continuum of 0–100 (Ulrich 2019). Equipment used for testing is shown in Figure 1-OA1, Online Appendix 1.

### Statistical analysis

All the data were reported using a commercially available software package (SPSS, Version 29.0, Inc., Chicago, Illinois, United States). Data were numerically coded, and no participant was identifiable from the data set. Participants’ TGMD-3 locomotor and ball skills subtest raw scores and overall gross motor performance raw scores were used as the dependent variables. Other studies conducting reliability analysis using the TGMD-3 instrument also analysed the raw scores, rather than standardised scores (Allen et al. 2017; Capio et al. 2016, 2018; Roczniak et al. 2025; Simons & Eyitayo 2016; Tun et al. 2023). Descriptive statistics were expressed as mean and standard deviations (s.d.s). The normality of the data was assessed with the Shapiro–Wilk statistical test and visually inspected with histograms and QQ-plots.

The feasibility of the tests was expressed as a percentage of completion rates. Differences between test and retest were analysed by a paired *t*-test or Wilcoxon signed-rank test for the locomotor and ball subtests as well as the total gross motor score. The test–retest relative reliability of data for all repeated tests was assessed with intraclass correlation coefficients (ICC) at 95% confidence intervals by using a one-way analysis of variance. This model treats all sources of measurement variation as an error, providing an accurate estimate of the stability reliability. The test–retest absolute variability was measured by using the standard error of measurement (SEM) to calculate the minimal detectable change at the 95% confidence interval (MDC_95_). The following formulae were used (Stratford 2004) SEM = Pooled s.d. × √(1 – ICC); MDC_95_ = SEM × 1.96 × √2.

The test–retest reliability was calculated between test 1 and test 2 of each analysis. The same principal investigator conducted all assessments but was blinded to previous scores. Intraclass correlation coefficient values of less than 0.50, between 0.50 and 0.75, between 0.75 and 0.90 and greater than 0.90 were classified as poor, moderate, good reliability and excellent reliability, respectively (Koo & Li 2016). The reliability data were also visualised through a Bland–Altman plot (Bland & Altman 1986). The difference between the two tests is plotted against the mean of the two tests for each participant.

Internal consistency was calculated using Cronbach’s alpha and was classified by George and Mallery (2003) as follows: > 0.9 – Excellent, > 0.8 – Good, > 0.7 – Acceptable, > 0.6 – Questionable, > 0.5 – Poor and 0.5 – Unacceptable for the coefficient alpha size. A *p*-value of less than 0.05 was considered statistically significant.

### Ethical considerations

Ethical clearance for this study was obtained from the Health Research Ethics Committee of the NWU Faculty of Health Science (NWU-00017-19-S1). Permission to conduct this study was obtained from the principals of the special needs schools from where participants were drawn. The Department of Education at the provincial level also granted permission to the researcher before the commencement of data collection. Even though the participants’ parents and guardians provided written informed consent before the study commenced, all participants were given an assent form to sign, which required them to select either a happy (green colour) or a sad face (red colour). The researcher obtained permission from the author of the TGMD-3 test manual to photocopy the examiner record form for data collection.

## Results

All study participants (*n* = 24), with a mean age of 11.2 years (±1.6) completed all gross motor performance tests (100% completion rate). Participants’ demographic profiles are presented in Table 1. All tests were safe and feasible, and no injuries were reported during the testing or retesting period.

Descriptive statistics are provided for mean, median and s.d. and the interquartile range (Table 2). The data were not normally distributed with respect to a visual inspection of the histograms, QQ-plots and an analysis of the Shapiro–Wilk test statistic.

**TABLE 2:** Descriptive statistics of the test and retest of Test of Gross Motor Development-3 subtests and overall gross motor performance for children with Down syndrome.

TGMD-3 Raw scores	Mean		Median		s.d.		IQR	
	Test	Retest	Test	Retest	Test	Retest	Test	Retest
Locomotor skills	4.63	4.46	4.00	3.00	3.33	3.81	3.00	3.00
Ball skills	6.58	6.38	6.00	5.00	3.16	3.45	3.00	5.00
Overall	11.21	10.83	9.50	8.00	6.05	6.99	4.00	8.00

IQR, interquartile range; s.d., standard deviation; TGMD-3, Test of Gross Motor Development-3.

The main finding of the current study, as displayed in Table 3, indicates that locomotor, ball and overall motor performance raw scores demonstrated excellent and good test–retest reliability coefficients (ICC’s > 0.8) in children with Down syndrome (*n* = 24). Standard error of measurement and values of minimal detectable change at the 95% confidence interval are also depicted in Table 3. Internal consistency demonstrated excellent and good alpha coefficients, as displayed in Table 3. The Wilcoxon signed rank test yielded no significant differences between the test and retest of locomotor skills (*p* = 0.587) and ball skills (*p* = 0.403).

**TABLE 3:** Test–retest reliability and internal consistency of Test of Gross Motor Development-3 subtests and overall gross motor performance for children with Down syndrome.

TGMD-3 Raw scores	ICC	95% CI	SEM	MDC_95_	Cronbach’s alpha
Locomotor skills	0.905	0.780–0.959	1.10	3.06	0.90
Ball skills	0.843	0.636–0.932	1.31	3.63	0.84
Overall	0.911	0.795–0.962	1.95	5.41	0.91

95% CI, 95% confidence interval; ICC, intraclass correlation coefficients; MDC_95_, minimal detectable change at 95% CI; SEM, standard error of measurement; TGMD-3, Test of Gross Motor Development-3.

**FIGURE 2:**
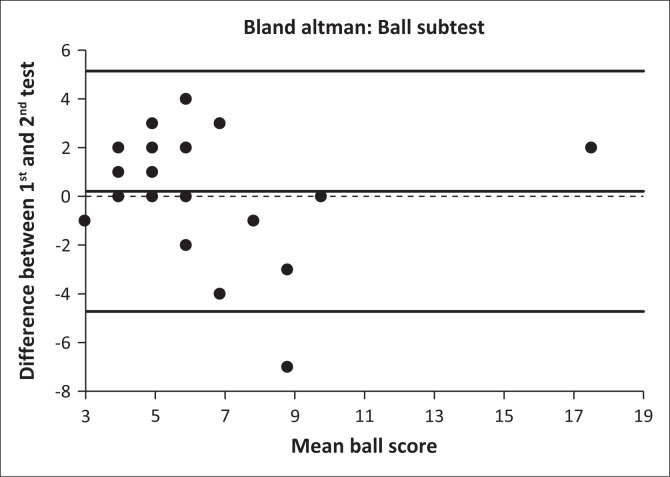
Bland–Altman plot for the ball subtest.

Figure 1 and Figure 2 contain the Bland–Altman plots for locomotor and ball skills test, illustrating the difference between the first and second test (*Y*-axis) against the participants’ mean of the two tests (*X*-axis). Each data point represents the difference between the two methods for each participant. The centre line indicates the mean difference between the tests, and the two outer lines indicate 2 s.d.s of the mean. The analyses suggest that there was no major systematic bias in the plots, although there were a few outliers. The scatter plot around the Bland–Altman was randomly distributed.

## Discussion

The primary aim of this study was to conduct the test–retest reliability of the TGMD-3 instrument amongst selected children with Down syndrome in South Africa. All participants were able to complete all 13 tests, and no injuries or accidents were reported from exercise testing for either assessment period. For the first time, a study has assessed the test–retest reliability of the TGMD-3 in an exclusive population of children with Down syndrome. Excellent and good ICC values were reported for locomotor (0.91), ball (0.84) and overall gross motor performance (0.91) in children with Down syndrome. The alpha coefficients for locomotor (0.90), ball (0.84) and overall gross motor performance (0.91) showed excellent to good internal consistency. Using the TGMD-2, two studies conducted on children with Down syndrome and intellectual disability also reported adequate internal consistency, with alpha coefficients larger than 0.82 for the locomotor, ball and overall gross motor performance (Simons et al. 2008; Tun et al. 2023).

Standard error of measurement, MDC_95_ and Bland–Altman plots showed acceptable precision, low variability, a marginally small discrepancy between scores for the two assessments and no systematic bias in the analyses. There were no significant differences between test and retest values for locomotor (*p* = 0.587), ball (*p* = 0.403) or overall gross motor scores (*p* = 0.321). Intraclass correlation coefficient values greater than 0.90 were also shown for the TGMD-3 instrument for studies conducted in typically developing children in the United States, Brazil, Germany and Iran (Mohammadi et al. 2019; Valentini, Zanella & Webster 2017; Wagner, Webster & Ulrich 2017; Webster & Ulrich 2017). Similarly, when using the TGMD-2 instrument in Belgium, values greater than 0.90 were also reported for non-Down syndrome children with intellectual disability (Simons et al. 2008). The only previous test–retest reliability study conducted on children with Down syndrome used the TGMD-2 reported ICC values of 0.92, 0.94 and 0.94 for locomotor, ball and overall gross motor scores, respectively, but the researchers reported significantly improved (*p* < 0.05) mean test values (> 4.0) for all locomotor, ball and overall gross motor scores from test 1 to test 2 (Tun et al. 2023). No explanation for this variation was provided, and SEM, MDC_95_ scores and Bland–Altman plots were not provided.

A possible reason for the high ICCs, low SEMs and random scatter of the Bland–Altman plots for the current study could be that test items with already desirable reliability and validity in other populations were used, including typically developing children (Webster & Ulrich 2017), children with autism spectrum disorder (Allen et al. 2017), children with visual impairment (Houwen et al. 2010) and children with intellectual disability (Simons et al. 2008). Other reasons could include that adequate practise and familiarisation sessions were performed to avoid the learning effect, and that concise and simple communication was used with study participants (Ulrich 2019). Few studies provided Bland–Altman plots and few listed error estimates, such as SEM. The error estimates of the test–retest reliability of the TGMD-3 of the current study (1.1–2.0) is lower than those reported by Allen et al. (2017) for children with autism spectrum disorder (2.2–4.1).

Minimal detectable change scores at 95% confidence interval reported in the current study should help to determine whether the change measured in future experimental studies is due to error or treatment. The magnitude of change after an intervention treatment should exceed the foreseen measurement error and variability. The current study reported an MDC_95_ value of 5.41 for overall gross motor functioning, and, thus, a value exceeding this amount would be needed to show significant improvement due to an intervention implemented for children with Down syndrome. Information of this nature could help to determine and monitor performance alterations over time as well as the success of training interventions, as reported in a recent study conducted on children with Down syndrome using the TGMD-3 (Staples et al. 2020).

The results of the current study show very low gross motor scores for children with Down syndrome, as corroborated in the study by Tun et al. (2023). It has been shown that children with Down syndrome perform less satisfactorily than children with intellectual disability and those with borderline cognitive impairment on locomotor and ball control tasks, as assessed through the TGMD-3 (Alesi & Battaglia 2019). Alesi et al. (2014) confirmed the association between motor and cognitive domains for individuals with atypical development. It could be speculated that the low gross motor scores could be related to the very low physical activity levels of children and adolescents with Down syndrome, as reported in a recent systematic review (Fox et al. 2019). It could also be related to high obesity levels (Bertapelli et al. 2016), cognitive impairment (Abd El-Hady, Abd El-Azim & El-Talawy 2018; Hartman et al. 2010), and the many health-related or physical impairments manifested in these individuals (Bergström et al. 2016; Capone et al. 2018; Foley & Killeen 2019). It has been reported that cognitive impairments may be associated with chronic medical conditions, confirming the interrelatedness of some of these factors as reported in a recent systematic review of children and young adults with Down syndrome (Gandy et al. 2020). Most individuals with Down syndrome have low muscle strength, muscle hypotonia, ligamentous laxity, reduced gait stability and poor coordination (Boer 2022; Carmeli et al. 2002; Mendonca et al. 2010; Terblanche & Boer 2013). All these factors could possibly contribute to a reduced gross motor proficiency. Until future, predictive, causative or associative studies confirm these speculations, these possible associations remain unproven. Some studies have provided evidence of associations between functional status and motor ability (Volman et al. 2007), cognitive function and gross motor skills (Abd El-Hady et al. 2018; Klotzbier, Holfelder & Schott 2022) and sensory processing function and gross motor skills (Brugnaro et al. 2024) for children with Down syndrome.

As suggested by Hartman et al. (2010), regular opportunities for free play, as well as teacher-directed training activities, could improve gross motor development. A recent 10-week physical education intervention study conducted in Texas, United States, demonstrated significant improvements (*p* < 0.01) in locomotor (+2.23) and ball skills (+2.23) with moderate to large effect sizes for children with Down syndrome (Staples et al. 2020). Moreover, an improvement in gross motor skills or performance has been reported for children with Down syndrome using therapeutic physical exercises (Popa & Dobrescu 2017), hippotherapy (Champagne & Dugas 2010), virtual reality therapy combined with physiotherapy (Stander et al. 2021), ‘Kashi practises’ (Kashi et al. 2015), Pilates exercises (Al-Nemr & Reffat 2024), adapted physical education and homework (Young 2017) and exercise training by coaches and family (Alesi et al. 2014; Ruiz-González et al. 2019). These interventions can be provided at a very young age for children with Down syndrome, as indicated in recent systematic reviews (Dumuids-Vernet et al. 2022; Rodríguez-Grande et al. 2022). As early as 2010, Hartman et al. (2010) emphasised the need for early intervention, seeing that motor skills and cognitive functioning are related and could be targeted with appropriate intervention strategies, as confirmed by Connolly, Morgan and Russell (1984). A longitudinal study confirmed the success of early intervention studies on motor performance for children with Down syndrome (Connolly et al. 1993).

### Limitations and future studies

A limitation of the current study is that the level of intellectual disability could not be determined, since the special needs schools did not have any information pertaining to the intelligence quotient of the participants. However, all participants in the current study fully understood the instructions and information pertaining to test procedures and techniques. Consequently, the results of the current study cannot be generalised to those with severe intellectual disability who cannot understand test instructions. It would be very worthwhile to analyse the level of intellectual disability as a cofactor in reliability studies. The results of the current study cannot be generalised to those with mosaic Down syndrome, since all the participants in this study had trisomy 21-type Down syndrome. Future studies could focus on those with mosaic-type Down syndrome (less than 2% of Down syndrome population). The final aspect related to generalisability is that the results of this study are based on one province in South Africa, consisting of a small sample size of 24 individuals; therefore, it may not be representative of the rest of the country or other countries.

A further limitation of the current study, as with all reliability studies, is the potential influence of a learning effect on subsequent administrations, whereby increased familiarity with the tasks may result in improved scores independent of true ability. Nevertheless, test–retest designs remain essential, as there are few practical alternatives for evaluating reliability over time. Importantly, the inclusion of adequate familiarisation sessions, together with the correct application of the TGMD-3 assessment protocol and the use of a trained assessor, may help to mitigate this limitation by reducing the impact of initial learning effects (Ulrich 2019).

Furthermore, future studies may need to determine the inter-rater and intra-rater reliability and validity, developmental sensitivity and clinical utility of the TGMD-3 instrument for children with Down syndrome, as recommended by Allen et al. (2017), who conducted their study on children with autism spectrum disorder. Test–retest reliability in isolation does not ensure the standardisation of an instrument. The sample size of the current study is small, and the researcher cautions against generalising the findings to all children with Down syndrome. Moreover, a sub-analysis or comparison of gender and age could not be conducted due to the small sample size.

## Conclusion

The findings in this study indicate that the TGMD-3 instrument yielded good to excellent test–retest reliability for assessing gross motor skills in children with Down syndrome. Whilst there are limited investigations to standardise gross motor skill instruments for children with Down syndrome, this study provides an initial reliability analysis of the TGMD-3 instrument. Future studies could confirm the inter-rater and intra-rater reliability of this instrument for use in this population. Furthermore, the test–retest reliability of this instrument remains to be determined for children with severe intellectual disability, who may not understand test instructions and demonstrations.
